# Linguistic Processing of Accented Speech Across the Lifespan

**DOI:** 10.3389/fpsyg.2012.00479

**Published:** 2012-11-08

**Authors:** Alejandrina Cristia, Amanda Seidl, Charlotte Vaughn, Rachel Schmale, Ann Bradlow, Caroline Floccia

**Affiliations:** ^1^Max Planck Institute for PsycholinguisticsNijmegen, Netherlands; ^2^Purdue UniversityWest Lafayette, IN, USA; ^3^Northwestern UniversityEvanston, IL, USA; ^4^North Park UniversityChicago, IL, USA; ^5^Plymouth UniversityPlymouth, UK

**Keywords:** infancy, childhood, aging, accent adaptation, speech perception

## Abstract

In most of the world, people have regular exposure to multiple accents. Therefore, learning to quickly process accented speech is a prerequisite to successful communication. In this paper, we examine work on the perception of accented speech across the lifespan, from early infancy to late adulthood. Unfamiliar accents initially impair linguistic processing by infants, children, younger adults, and older adults, but listeners of all ages come to adapt to accented speech. Emergent research also goes beyond these perceptual abilities, by assessing links with production and the relative contributions of linguistic knowledge and general cognitive skills. We conclude by underlining points of convergence across ages, and the gaps left to face in future work.

## Introduction

Infants, children, and adults may all experience different challenges in processing unfamiliar accents. Learning how to adapt to unfamiliar accents is necessary for efficient language processing, given the variety of accents that surrounds us. Nearly everywhere in the world, a simple trip to the market will most likely put you within earshot of dialectal or foreign accents. For instance, a report of 26 countries by the Organization for Economic Cooperation and Development (2007) estimated that about 9% of each country’s population was foreign and thus might speak a language not spoken in their current country of residence. To take a more specific example, a census report in the USA documents that 20% of respondents declared speaking a language other than English at home, and half of that 20% estimated their own English speaking abilities as below fluent (United States Census Bureau, 2008). Moreover, these numbers underestimate the likelihood of encountering an accent different from one’s own, as they do not take into account variation in within-language accents. In this article, we review evidence bearing on how we perceive speech in the face of accent variation, both as our linguistic system develops and after we have become efficient language processors. To our knowledge, this is the first review that aims to assemble findings on infant, child, and adult accent perception. Examining accent perception across the lifespan allows us to underline points of convergence and divergence, as well as gaps that remain for future work.

Before we delve into this literature, we clarify a few terminological points. First, we use the term *linguistic variety* as an umbrella covering different (1) regional or sociolectal varieties of a single language (i.e., *within-language* accents), (2) different languages, and (3) non-native varieties of a language (i.e., *foreign* accents). In addition, we define the term *accent* from the listener’s perspective, as follows: a talker may be described as accented if his/her speech diverges from that of the listener’s systematically at the suprasegmental and/or segmental level. Consequently, if the listener speaks a “non-standard” regional variety, and the talker a standard variety, the latter would still be described as *accented*, because his/her speech deviates from that of the listener. Notice that we extend Wells’ (1982) definition of accent, as deviations along the phonetic, phonotactic, phonological, and lexical levels, in order to encompass the suprasegmental level as well.

In the following four sections, we summarize current literature on accent perception in young adults, infants, children, and older adults. Looking throughout all age groups, we identified two central themes of research evident in each and every age group. One theme pertains to initial processing difficulties exhibited when hearing accented speech; the other to the effects of exposure to the accent. Each of the following four sections is devoted to research on one age group, reviewing research on each of the two themes, and ending with a summary and brief discussion of the particular contributions of that age group to our understanding of accented speech perception. Research on young adults is presented first because the majority of research on accent perception has been carried out on young adults, typically college students. Furthermore, work with other populations usually use young adults as a reference point. Thus, these data can be viewed as the benchmark against which researchers working with younger or older populations will compare their findings.

## Accent Perception in Early Adulthood

In view of the wealth of this work, we do not endeavor to be comprehensive in the present section, but focus instead on lines of research that allow comparisons with the literature from other age groups summarized below. The interested reader is referred to Floccia et al. (2006) and Samuel and Kraljic (2009) for more detailed reviews focusing on adult perception of accented speech. Here, we limit our summaries to a few representative studies documenting both the initial processing costs and the adaptive processes involved in recognizing the linguistic content of accented speech.

### Initial processing costs

The rich work on accented speech perception that has been carried out on young adults shows that accented speech affects both accuracy and speed of processing. Listeners are less accurate in transcribing the speech of both foreign accented speakers (Gass and Varonis, 1984) and within-language accented speakers (Mason, 1946; Labov and Ash, 1997). Moreover, intelligibility of both foreign accented speech (Rogers et al., 2004) and regional accented speech (Clopper and Bradlow, 2008) can be affected by background noise to a greater extent than speech spoken in the listeners’ own accent. Accented speech is also processed more slowly. This has been shown in a wide range of tasks, including assessing whether a wordform is a real word or not (Floccia et al., 2006), deciding whether a word heard matches one printed on a screen (Munro and Derwing, 1995; Clarke and Garrett, 2004), making semantic judgments (Adank and McQueen, 2007), and evaluating whether a sentence is true or false (Adank et al., 2009).

In fact, some research suggests that delays when processing speech in an accent that is not one’s own could actually indicate that different mechanisms are recruited, or that they are relied upon to a different extent when processing accented and unaccented speech. These differences are sometimes evident when processing is rendered difficult. For example, Bürki-Cohen et al. (2001) tested native English listeners on a phoneme detection task, either in isolation or paired with a secondary linguistic task (deciding whether the item was a noun or a verb). The key question was whether listeners would in fact recruit lexical information in their judgments, in which case response times should be lower for higher frequency words than for lower frequency words. For the unaccented speech, listeners did not make use of lexical information (response times did not vary between high and low-frequency words), even when the secondary task was added. However, the secondary task led listeners to rely on lexical information when processing foreign accented speech.

Interestingly, several top-down factors have been shown to modulate the processing cost involved in perceiving accented speech, suggesting that, to a certain extent, a different processing profile may not be due only to differences in the acoustic signal. Indeed, the mere expectation that speakers will have an accent may hinder listeners’ comprehension. For example, Rubin (1992) found that the same general American “unaccented” speech was understood less accurately when paired with a photograph of an Asian face than when it was paired with a Caucasian face. Although it remains to be explored to what extent such top-down factors impact lower-level processing, it is clear that the challenges involved in processing accented speech go beyond the fact that listeners’ usual mechanisms are suboptimal in the face of less familiar speech patterns.

### Effects of exposure

In order to have precise control on exposure and accent complexity, a recent line of work has opted to train listeners on artificially created, novel accents. These studies simulate what it is like to be exposed to a new regional or foreign accent – or at least to one feature of a new accent – and thus may help us better understand how initial adaptation to real accents might occur. For example, Maye et al. (2008) created an accent where all vowels were shifted down in the vowel space (i.e., “wetch” became an acceptable pronunciation of the word “witch”). After mere minutes of hearing the story of the Wizard of Oz spoken in this “accent,” participants gave more “word” responses on a subsequent lexical decision task to items that were plausible implementations of real words in that accent. Most work shows that adult listeners adapt to novel pronunciations by applying top-down, lexical knowledge (Norris et al., 2003; see Mitterer and McQueen, 2009, for evidence that orthographically presented forms may also facilitate adaptation); but reports also exist for other sources of disambiguation, such as visual input (Bertelson et al., 2003) and phonotactics (Cutler et al., 2008).

There is considerable debate surrounding the format of adaptation. Some argue that changes occur at a prelexical level of interpretation, since the transfer to untrained lexical items is cost-less (e.g., Maye et al., 2008). Along a similar line, Skoruppa and Peperkamp (2011) argue that such adaptation crucially recruits phonological knowledge, since it is constrained by phonological simplicity. In their study, listeners were able to learn an accent in which vowels harmonized (i.e., vowels in adjacent syllables had to both be rounded – the French word *liqueur/*likœʁ/was pronounced *luqueur/lykœʁ/, where the first vowel has become rounded) or disharmonized (*pudeur*/pydœʁ/became *pudere/pydεʁ/, with the second vowel becoming unrounded). However, listeners failed to encode an accent that was unnaturally complex (where mid vowels harmonized and high vowels disharmonized). Others argue that adaptation involves altering lexical representations, for example storing the unusual instantiations without abstracting away talker information. Dahan et al. (2008) argue for this interpretation based on effects in lexical competition. They measured lexical competition by tracking eye movements to potential visual referents of minimally differing words, such as “back” and “bag,” while one of these words was presented auditorily. For half of the participants, the talker in the stimuli raised/æ/to [ε] before/g/; for the other half, she did not. When hearing the beginning of the unaltered word “back,” participants in the latter (control) group sometimes looked at the picture of “bag,” showing a certain amount of lexical competition between the two items. When faced with the same situation, the group exposed to raised /æg/ words looked less to “bag” than the control group did. In other words, the raised group found /bæk/ less ambiguous between the “bag” and “back” interpretations (i.e., if the talker meant to say “bag,” the syllable should have started with /bε/). Such artificial phonologies are well-suited to investigate the question of how we encode accented speech patterns.

However, natural accents are much more complex than those previously implemented through artificial phonologies; for example, the former typically affect multiple phonological levels. Research is still needed to assess the extent to which learning such artificial phonologies resembles learning real accents. Thus, this work cannot currently replace research investigating the potential benefits of short-term, laboratory-based exposure to a natural accent, which provides valuable evidence. For example, Clarke and Garrett (2004) showed that response times for foreign accented speech in a cross-modal matching task (assessing whether a word heard and a word printed on the screen are the same) can catch up with the native accent response times after less than a minute of exposure (see also Wingstedt and Schulman, 1987, for transference to production, and Kraljic et al., 2008a, for adaptation in perception without transfer to production). However, how and when exactly this adaptation can be brought about in the lab is not entirely clear.

Bradlow and Bent (2008) provided important evidence concerning when accent adaptation is more likely to occur. Specifically, they found that exposure to multiple Chinese-accented speakers improved adaptation to a novel Chinese-accented speaker to a larger extent than exposure to a single Chinese-accented speaker did. Different groups of native English listeners heard sentences in noise produced either by five unaccented speakers, the same Chinese-accented speaker who would serve as the test speaker, one Chinese-accented speaker who was *not* the test speaker, or five Chinese-accented speakers, and were asked to transcribe sentences played to them. Following this exposure phase was another sentence transcription task serving as a test. Participants who heard one Chinese-accented speaker in training and a different Chinese-accented speaker at test did not perform any better than participants who heard unaccented speakers in training. In contrast, exposure to multiple Chinese-accented talkers resulted in adaptation to a novel Chinese-accented talker, at a level equivalent to being trained with the test talker. Thus, it seems that exposure to multiple talkers of the target foreign accent can be an effective means of achieving talker-independent adaptation in adults. Interestingly, this adaptation was accent-dependent rather than accent-general since training on Chinese-accented English (whether with one or five talkers of the accent) did not result in adaptation to another unfamiliar accent (Slovakian-accented English).

Other work focuses on the effects of long-term exposure. For example, native speakers of British English show less difficulty processing American English speakers’ productions of medial-/t/as a tap (“ciddy” for “city”) if they have lived in the United States (Scott and Cutler, 1984). Sumner and Samuel (2009) studied the processing of r-final words spoken by talkers of rhotic dialects (General American English/GA), who pronounce the final/r/, or non-rhotic dialects (New York City English/NYC), who do not pronounce the final /r/ (“bakuh” for “baker”). This study used a priming paradigm and tested listeners who were either familiar or not with NYC English. For all participants, a target was never primed as well if it had a prime of a mismatching dialect, showing an overall cost of switching dialects from prime to target. Further, participants with prior exposure to NYC English showed both form (“slenda” primes “slenda” and “slender”) and semantic priming (“slenda” primes “thin”) for NYC English primes on NYC English and GA English targets. However, GA English speakers did not show semantic priming for NYC English primes (“slenda” does not prime “thin”), suggesting that experience with the dialect is necessary for a dialect form to facilitate processing. Exposure effects are also evident when exposure is mostly through media, as shown by studies of asymmetrical cross-accent perception. For example, Impe et al. (2008) found that Netherlandic speakers of Dutch are much slower in a lexical decision task when processing words recorded by Belgian Dutch speakers than by fellow Dutchmen, while Belgian Dutch speakers process both varieties equally well. Similarly, in Adank et al. (2009), a truth value judgment task was administered to both Glasgow and London listeners using spoken stimuli recorded from Glasgow and London talkers. While Londoners were slower with the speech from Glasgow speakers than the speech from speakers of their own accent, Glaswegians were equally fast with both accents. These asymmetries fit with asymmetries in media exposure of the two accents.

A combination of behavioral and electrophysiological measures begins to shed light on the prelexical and lexical effects of a lifetime of exposure to multiple linguistic varieties on the perception of native contrasts, as well as those present in non-native, but familiar, varieties. This question has been approached using regional variation in French. The varieties spoken in France have either lost or are in the process of merging /e/ and /ε/, a contrast that has not merged in the varieties spoken in Switzerland. Current results indicate that long-term exposure to a variety where a given contrast is merged (i.e., French as spoken in France) could actually result in loss of discrimination in one’s own unmerged variety (affecting Swiss listeners; Brunellière et al., 2009, 2011). Additionally, being exposed to a variety which has a contrast where one’s native variety has merged does not suffice to preserve baseline discrimination (Dufour et al., 2007), nor typical lexical access (Dufour et al., in press); and discrimination training on the preserved contrast does not result in normal lexical processing either (Dufour et al., 2010).

Pure exposure, however, is not the only factor affecting accent processing. In the semantic priming study discussed above, Sumner and Samuel (2009) also reported that participants who were familiar with the NYC dialect but were not non-rhotic in their own productions did not retain the priming benefit after a 20–30 min lag, while participants who were non-rhotic in their own production *did* show long-term priming. Similarly, Kendall and Fridland (2012) report that individual listeners from various regions of the USA categorized vowels along an /e/-/ε/ continuum differently depending on not only the listener’s region of origin, but also their own production of those sociophonetically marked vowels. The correlations between production and perception can even be tracked longitudinally. Evans and Iverson (2007) collected production and perception measures over a period of 2 years from students who had moved from northern England to attend college in southern England. Results showed that students who displayed more southern English accent features in their own speech also exhibited better adaptation to southern speech in perception tasks.

Naturally, it is impossible to know whether such correlations are mediated by the quality of experience in terms of perception only versus production-perception, or whether the social values and/or experiential opportunities that accompany a given speech pattern also play a role (Labov, 2007). That is, one can posit many explanations: perhaps these correlations are caused by amount of exposure to the preferred accent (e.g., people who adopt the local accent are precisely those who spend more time with locals), or by the social valence attached to accent adaptation (e.g., people who show incipient traces of adaptation in their production get more positive reinforcement from locals, which further boosts their perceptual adaptation). In a recent laboratory study, the causal link between production and ease of processing has been documented: Adank et al. (2010) evaluated ease of processing (as measured in terms of the maximal tolerated signal to noise ratios) of a relatively unfamiliar accent after different types of training. In some, listeners simply had to listen; in others, they were asked to repeat, or write down the words they heard; in yet others, they were asked to repeat imitating the accent. Significant improvements were only evident when the training involved imitation of the novel accent.

Interestingly, our knowledge of regional accents shapes our perceptual expectations. In fact, merely evoking a specific region – through the name of a place being written on a response sheet (Niedzielski, 1999) or through exposure to a stuffed toy that brings into mind a particular place (Hay and Drager, 2010) – causes listeners who are familiar with the dialects of both regions to categorize vowels in accordance with the evoked region’s accent pattern. The shifting of phoneme categorization boundaries seen in these studies reflects adaptation to the incoming speech signal, contingent upon the listener’s knowledge of the patterns of a particular within-language accent.

### Summary

This brief overview of accent perception research in young adults has allowed us to identify a few key findings, which will be carried over in our review of the developing and older populations. Accented speech initially perturbs word recognition and/or sentence processing in terms of accuracy (e.g., Gass and Varonis, 1984) and speed of processing (e.g., Floccia et al., 2006). With exposure in lab conditions, evidence of adaptation can be found (e.g., Clarke and Garrett, 2004). A lifelong exposure to a variety of accents shapes perceptual abilities so that listeners are able to process each variant equally rapidly (e.g., Sumner and Samuel, 2009), suggesting certain flexibility of the representations or the way the signal is mapped onto them. Finally, processing ease varies with factors that go beyond simple exposure (e.g., Kendall and Fridland, 2012).

## Accent Perception in Infancy and Toddlerhood

Before embarking on this section, we remind readers of the unique challenges of research with very young children who cannot respond to explicit instructions. Despite this challenge, there is one considerable empirical advantage to working with infants and toddlers: it is much easier to gage, and at times even measure, the linguistic input participants have had over the course of their relatively short lifespan. As a result, studies on infants and toddlers can shed unique light on the effects of exposure. It should also be mentioned that this is a burgeoning research area, and that some of the questions set out here have already begun to be answered.

### Processing costs

In accent discrimination studies, infants are familiarized with one linguistic variety until they accumulate a certain amount of “attention time.” Then in a subsequent test phase, infants are presented with two types of trials, one in which the familiarized variety is presented, and another in which a novel variety is presented. Presentation of the sound is contingent on the infant orienting toward the source of the sound, and looking times are measured. Discrimination is surmised to have occurred if infants orient longer when hearing the novel variety than when the familiarized variety is being presented. Under these conditions, 5- and 7-month-olds can *only* discriminate rhythmically similar linguistic varieties if they have experience with at least one of the varieties on which they are tested; that is, they can discriminate their native variety from an unfamiliar one, but not between two unfamiliar, rhythmically similar linguistic varieties (Nazzi et al., 2000; Butler et al., 2011).

Preference paradigms skip the familiarization phase to tap infants’ early preferences for one variety over another, simply measuring infants’ attention while they hear utterances in their own or an unfamiliar variety. In this paradigm, preference is dependent on age (younger infants show stronger preferences than younger ones), and experience (infants with some exposure to the non-native variety lose their preferences earlier; Kitamura et al., 2006). A decrease of preference for the native over the non-native variety has been taken as evidence that infants learn to interpret the unfamiliar accents as a variant of the native accent. In other words, younger infants fail not because they are unable to implement compensatory strategies to “correct” for the accent, but rather because they reject the accented speech as a potential implementation of their native language. However, an alternative cognitive interpretation is that these early preferences are driven by ease of processing. For example, it may be that young infants have a difficult time processing unfamiliar variants, and thus implicitly dislike the non-native variant (this is a possibility that we discuss in greater detail in See Concluding Remarks). As they age, their processing abilities may expand (e.g., vocabulary size, working memory, selective attention), and thus they may find it easier to process even the unfamiliar properties of accented speech. This would occur in the domain of accent processing similar to the way it occurs in other cognitive domains that involve processing at more than one level. For example, as working memory increases, the child is able to process both the social valence of an accent (Kinzler et al., 2007), and its linguistic content simultaneously. The two explanations differ only in whether age changes reflect variation in metalinguistic judgments (i.e., as the infant ages, she learns that a language may have different accents, and all of them should be accepted), or variation in executive functions (i.e., as the infant ages, she becomes better at coping with difficult tasks). These alternatives could be explored by simplifying the task for younger infants (for example, presenting a single word, which reduces memory load), or by increasing processing demands in older infants (e.g., through the addition of a concurrent task). If either of these manipulations reverts previous preference patterns (disappear in younger infants, reappear in older infants), then processing resources (rather than a metalinguistic reinterpretation of language varieties) would be implicated.

Beyond this issue of interpretation, it is clear that young infants are sensitive to the acoustic changes introduced by different linguistic varieties. Consequently, one may wonder whether infants are able to segment wordforms when suprasegmental and subphonemic cues are altered by accented speech. This is important because other research suggests infants are very sensitive to acoustic mismatches in the wordforms they hear (Singh et al., 2004). For example, infants sometimes fail to recognize words across very different voices (Houston and Jusczyk, 2000; but see van Heugten and Johnson, 2012). In a typical segmentation task, infants are first familiarized with a wordform (e.g., they hear the word “candle” produced in isolation several times). At test, they are presented with several passages, only half of which contain the familiarized wordform, and the presentation of the passages is contingent on infants’ attention. When the same voice, speaking in the infants’ native accent, is used in familiarization and test, even 7.5-month-olds reliably prefer passages containing the familiar wordform (Jusczyk and Aslin, 1995). This is already no small feat, given that word segmentation is an incredibly complex task, as there is no single reliable cue to word boundaries in all languages. Interestingly, Polka and Sundara (2012) report that Quebecois French-learning 8-month-olds are as capable of segmenting words from unfamiliar European French as they are from their native variety, but they fail to segment Canadian English (which is also unfamiliar to them).

These results could suggest that 8-month-olds can already accommodate for within-language varieties, in a way that does not extend to an unfamiliar language. However, we believe this interpretation is too strong in view of the following two sets of results. First, infants raised in Paris show no behavioral evidence of segmenting words from their own native variety until several months later (Nazzi et al., 2006), yet these European French-learners succeed in segmenting the unfamiliar Canadian French variety at 8 months (Nazzi et al., 2012). Further, American English-learning 9-month-olds are able to segment words in Dutch, a language unfamiliar to them (Houston et al., 2000). Segmentation in these studies could be resolved through some approximate pattern-matching that does not require extensive experience with the specific linguistic variety, and it could be affected by a host of acoustic and prosodic factors that are difficult to control across varieties (see e.g., Singh, 2008, for evidence of the impact of non-linguistic variation in such tasks). For example, Nazzi et al. (2012) propose that irrelevant prosodic cues (e.g., the quality or degree of the infant-directed speech and hence its likability) could shape infants’ performance when not explicitly and carefully controlled.

Cross-accent segmentation studies ask whether infants can recognize and segment a familiarized word across the native variety and an accented variety. Specifically, the procedure is identical to the segmentation studies described above, except that the familiarization stimuli are spoken in one accent, and the test passages in a different accent. Using this design, Schmale and Seidl (2009) tested 9- and 13-month-olds on their tolerance for a change between the native and a foreign (Spanish) accent, whereas Schmale et al. (2010) assessed accommodation across two within-language English accents (North Midland American and Southern Ontario Canadian) in 9- and 12-month-olds. In both cases, the older group succeeded where the younger group failed. Naturally, as we pointed out for the language preference tasks, here the effects of experience and maturation are confounded. Thus, one possibility is that older infants are better at segmenting words because most of them have accumulated more experience with diverse talkers, which allowed them to develop linguistic strategies to cope with talker variability. But there are many other alternatives; to give just one example, they may have also undergone experience-independent advancements in cognitive skills recruited by the task, such as selective attention and working memory. These advancements could be clearer when processing accented speech because infants are not at ceiling in that task.

Perhaps stronger evidence for accommodation to a novel accent is provided by word preference studies. In this paradigm, experimenters measure toddlers’ preference between two types of trials: trials with high-frequency wordforms, which are likely to be familiar to them (e.g., bottle); and trials with low-frequency wordforms, which toddlers have likely never heard (e.g., enzyme). In both kinds of trials, wordforms are presented auditorily, without any visual support, and attention is gaged by making the presentation of the speech contingent on the infant response. Infants as young as 11 months show a strong preference for high-frequency wordforms when tested in their familiar language variety (Hallé and de Boysson-Bardies, 1996). However, 15-month-olds growing up in Connecticut, USA showed no such preference when tested in an unfamiliar variety (Jamaican English), but they did when tested with their native variety, whereas 19-month-olds showed a high-frequency preference within both native and unfamiliar varieties (Best et al., 2009). This high-frequency preference paradigm offers a promising avenue of research, since it could shed light on toddlers’ long-term representations. Nevertheless, one area that needs to be explored more carefully is what exactly underlies these preferences. The standard interpretation is that toddlers are matching the wordforms they hear with their own long-term *lexical* entries. However, it is also possible that toddlers are evaluating these items *phonologically* only, since the contrast between high- and low-frequency wordforms has typically confounded the frequency of sounds and phonotactics.

Up to this point, we have discussed how infants and toddlers recognize wordforms in unfamiliar linguistic varieties. But a key point in accent accommodation is how we come to realize that two different pronunciations map onto the same *referent*. This topic has been breached using intermodal preference paradigms, where a wordform is heard at the same time that two pictures are displayed in the visual modality. Typically, toddlers will look longer at the target (the picture matching the wordform heard) than the competitor (another image on the screen that does not match the wordform). This matching preference ensues provided that the wordform is sufficiently similar to the target’s name to prime this association, that is, even when it is not identical (Swingley and Aslin, 2000). In Mulak et al. (in press), it is reported that 15-month-olds showed a significant preference for the matching picture when the wordform played was produced in their native variety (Australian English), but not in an unfamiliar one (Jamaican English), suggesting that the unfamiliar pronunciation departed significantly from the one toddlers were accustomed to processing. Similarly, unfamiliar accents may prevent recognition of newly learned words. Schmale et al. (2011) taught toddlers a new word by pairing a wordform with a picture. Then they were tested on their recognition of that word in two subsequent trials involving changes in language varieties. In one test trial, two pictures were displayed on the screen while the familiar wordform was provided (looks to the matching target are expected if children have learned the word-object association). The second test trial was more cognitively demanding, since a *new* label was provided, and toddlers were expected to infer that the correct referent was the competitor. In this demanding task, 30-month-olds were able to recognize a newly learned word across Spanish-accented and native English pronunciations, regardless of which variety was used in training and test. Contrastingly, 24-month-olds showed significant preferences for the object that matched the label (the trained object, when the trained label was provided; the novel object otherwise) when trained with a Spanish-accented talker and tested with a native English talker, but not when the opposite presentation order was provided. This order of presentation effect suggested that even short exposures to the accent could suffice in easing children into the unfamiliar accent, a possibility that was investigated in a study reported in the next section.

### Effects of exposure

White and Aslin (2011) examined the effects of exposure to an accent on toddlers’ accommodation of an unfamiliar variety using lexical feedback. Specifically, during a training phase, 19-month-olds saw pictures of highly familiar objects (e.g., block, bottle) while hearing the vowel in the words associated with that object consistently produced with an (æ) sound (as “black, battle”). At test, toddlers evidenced generalization of the consistent sound change to untrained, highly familiar words. For example, they looked longer to a picture of a sock (than to a picture of an irrelevant item) while hearing the word “*sack*,” but not when hearing the word “*sick*,” showing that the sound reinterpretation was relatively precise. Thus, 19-month-olds can adapt to novel accents when provided with clear and sufficient evidence.

Other work suggests that toddlers also benefit from more naturalistic exposure to a complex accent Schmale et al. (2012) exposed toddlers to brief stories with no accompanying visual referent. Thus, no effort was made to train toddlers on the host of phonetic changes imposed by a natural Spanish accent. After 2 min of exposure to such speech, 24-month-olds were able to recognize a newly learned word across their native accent and the foreign accent. Their performance was improved both when the same speaker was used for pre-exposure and test, and when four different voices with the same accent, none of whom produced the test stimuli, told the brief stories. Toddlers’ performance in accommodating the foreign accent was unaffected by a pre-exposure to one or four native English speakers, suggesting that the improvement was truly driven by foreign accent exposure.

These recent training studies suggest that even brief exposure can reshape infants’ perception of unfamiliar linguistic varieties of speech. A natural follow-up question is how long-term exposure to multiple varieties affects early development. One intriguing study suggests that bi-varietal toddlers recognize words better in the variety that is more widely spoken in their general environment, even if they have greater exposure to the minority form (Floccia et al., 2012). Word recognition was assessed in 20-month-olds growing up in a region where rhoticity was prevalent (e.g., “car” pronounced with a final “r” by most of the population). There were two groups of participants. One was a mono-varietal group, where both parents produced rhotic variants, as in the local environment. The other group was bi-varietal, because they were exposed to the locally predominant rhotic variant outside of the home, and they were exposed to a non-rhotic variant at home as one or both parents spoke a non-rhotic variety. Estimation of their weekly exposure showed that, overall, the bi-varietal children had more exposure to the non-rhotic variant (through one or both caregivers) than the rhotic variant (through the rhotic caregiver if they had one, plus the local context). All toddlers were tested with three groups of words: control items (lexical items that contained no “r”; produced by non-rhotic talkers), rhotic items (lexical items that contained “r” and had been produced by rhotic talkers), and non-rhotic items (lexical items that contained “r” but had been produced by non-rhotic talkers, and therefore there was no /r/ in them). Results revealed that both mono- and bi-varietal toddlers looked at the target significantly longer for both control and rhotic items, but neither group looked above chance for non-rhotic items. Thus, it appears that 20-month-olds are particularly sensitive to the frequency of forms across the local population. Notice in particular that the bi-varietal toddlers still performed better with the rhotic variant, despite their personal larger frequency of exposure to the non-rhotic forms.

### Summary

Before the age of 6 months, infants seem to have learned accent-specific information about their most commonly encountered variant that allows them to discriminate and prefer it to unfamiliar variants (e.g., Kitamura et al., 2006). Whereas infants’ and toddlers’ linguistic processing is impeded by an unfamiliar accent early on, they become better able to recognize words and wordforms across different accents with further development (due to changes in maturation and/or experience; e.g., Schmale and Seidl, 2009). Even holding maturation constant, brief exposure enables toddlers to cope with accent changes (e.g., Schmale et al., 2012). Finally, toddlers are better able to recognize words produced in the linguistic variety spoken widely in their local community, even when they primarily hear another variety at home (Floccia et al., 2012).

Thus, results reviewed largely coincide with the picture found in young adults, in that there are initial processing costs when a novel accent is encountered, which are diminished through brief exposure. However, results in infants and young adults do not align with respect to long-term exposure to multiple accents. In adults, a lifetime of exposure to an accent provides listeners with the ability to access the same lexical items through both varietal forms; for example, Sumner and Samuel (2009) document priming across regional variants. In contrast, the only study carried out so far with toddlers suggests that they do not store all variants encountered, nor do they attend more to those that are heard more frequently, but focus on variants that are more prevalent in their environment (Floccia et al., 2012).

An important contribution of this literature relates to the marked developmental changes that have been documented, with the general pattern being that older age groups succeed in recognizing wordforms across accents more readily than younger ones. One interpretation of such findings is that experience teaches learners to ignore accent-related differences, allowing them to retrieve the constant phonological and lexical information. Indeed, this is the viewpoint commonly adopted when documenting developmental changes in initial processing costs (e.g., Best et al., 2009). Another interpretation is that developmental changes are not tied with an awareness of what kinds of variation are to be ignored, but rather with changes in cognitive flexibility or executive control. For instance, one could postulate that advances in selective attention, memory, and executive control allow toddlers to more readily process accented speech in terms of both linguistic and social dimensions. The role of multiple cognitive and linguistic factors in accent processing and perceptual adaptation is a topic that we revisit in Section “Accent Perception in Late Adulthood”.

A second key contribution of the infant literature pertains to the mechanisms triggered by exposure to a novel accent. The disappearance of initial costs with exposure can occur in the absence of a large lexicon (as even 12-month-olds can recognize newly trained wordforms across accents; Schmale et al., 2011), and the effects of exposure to an accent are evident even without explicit lexical training (Schmale et al., 2012). We believe that the contribution of non-lexical factors to accent perception and adaptation is relatively understudied, as it is generally assumed that accent accommodation is primarily guided by lexical entries (there are a handful of exceptions, such as the studies cited above by Bertelson et al., 2003 and Cutler et al., 2008). The questions of how and whether listeners of different ages cope with accentual variation at phonological levels where lexical feedback is irrelevant (e.g., intonation) could be investigated in future work.

## Accent Perception in Childhood

Accent perception during childhood is less well-documented than accent perception in early infancy or in adulthood, possibly because the focus of many studies with children has been on production. Indeed, numerous questions within this topic have been studied, such as when children acquire local features of their native variety (Labov, 1989; Roberts and Labov, 1995; Roberts, 1997a,b, 2005; Jacewicz et al., 2010) or even a new language (Flege, 1999, 2003; DeKeyser, 2000) and to what extent they manage to acquire an accent in the local variety of a region they move into (Tagliamonte and Molfenter, 2007). Accent production research in children suggests an outstanding ability to acquire a new accent (e.g., Tagliamonte and Molfenter, 2007), which very likely suggests an excellent perceptual flexibility for accent variations. Some work argues that foreign accent in caregivers is ignored (in order to acquire the local native accent; Chambers, 2002). One line of research within perception has thus studied potential differences in the detection of native and foreign accents.

This line of work builds on research on young adults, who are highly adept at rating the accentedness of talkers (e.g., Piske et al., 2001), identifying and discriminating accented talkers (e.g., Goggin et al., 1991; Winters et al., 2008; Perrachione et al., 2009), and categorizing accented talkers according to regional language background (e.g., van Bezooijen and Gooskens, 1999; Clopper and Pisoni, 2004a,b; Clopper and Bradlow 2008). Children have also been tested on their ability to categorize talkers on the basis of their accents. Girard et al. (2008) presented French-speaking 5- to 6-year-olds with sentences in two accents and instructed them to group the speakers into two sets according to their accent. Results showed that children succeeded in this task only if the unfamiliar accent was a foreign accent, and not a within-language accent. Additional experiments showed that children were able to hear the within-language accent differences in a simple discrimination task, albeit to a lesser extent than foreign accent differences. This was interpreted as evidence that children were more sensitive, or aware of, unfamiliar foreign accents than of unfamiliar within-language accents. However, the perceived strength of the accent in the foreign accented stimuli was stronger than in the within-language accent ones in that study, which could have explained the greater salience of the foreign accented features than the within-language features. Floccia et al. (2009) addressed this concern by selecting stimuli spoken in a regional (Irish) accent and a foreign (French) accent on the basis of similar ratings of accent strength by British speakers of English. They then presented these items to 5- and 7-year-olds in a simpler version of the sentence categorization task, in which children were simply asked to spot the speaker who “spoke like an alien.” Results were similar to those of Girard et al. (2008) in the older group of children. Specifically, 7-year-olds were better at spotting the foreign accent over the within-language accent. Curiously, this same tendency was not statistically significant for the 5-year-olds. One interpretation of these results is that children are increasingly sensitive to a foreign accent with age and experience. More generally, this perceptual work demonstrates that children can perform metalinguistic tasks that require a reliance on accent.

### Initial processing costs

In Nathan et al. (1998), Londoner children were presented with isolated words produced in either a London or a Scottish Glaswegian English accent. After hearing a short story spoken in an accent, 4- and 7-year-olds heard words in isolation and were asked to report what word the talker had said, and to define it (notice that, since there was no control group who did not hear the story, we cannot shed light on the effects of exposure and thus do not include this paper in the next section). Responses were classified as phonological if the child correctly identified the Glaswegian-accented word, repeating it in her own London accent, or phonetic if the child imitated the form in the Glaswegian accent (and thus produced a form that was not a word in the London accent). As expected, accuracy in the definition task increased with age: older children gave significantly more phonological responses than phonetic ones, showing that they “corrected” the accented input. Alternatively, younger children gave significantly more phonetic responses than older ones. Enhanced phonetic sensitivity at 4 years (as compared to 7 years) might lead to enhanced adaptation abilities, which in turn would lead to an enhanced capacity to learn a new accent in production. Of course, another interpretation of these results is that younger children’s remarkable sensitivity to phonetic contrasts prevented them from accessing stored representations, as if acute phonetic abilities were blocking lexical accommodation. In sum, the Nathan et al. (1998) study provides the first evidence of developmental changes in within-language accent perception in childhood, documenting an improvement in children’s ability to identify common words across within-language accent variations between the ages of 4 and 7 years. However, the presentation of isolated words in the absence of context might have been partially responsible for the younger children’s inability to retrieve the intended word produced in the unfamiliar accent, whatever the underlying explanation. Therefore, studies using continuous speech sequences, or a greater guiding context, may provide a more realistic view of how children cope with unfamiliar accents in their everyday life. With this goal in mind, Holtby (2010) presented 9- and 15-year-olds with foreign accented sentences and asked them to assess the truth value of the sentence, to rate the intelligibility of the sentence, and to rate the accent strength of the sentences. In this study, there was no developmental difference in detecting a foreign accent, as shown by similar accentedness ratings in the 9- and 15-year-olds and similar intelligibility ratings. Although a significant improvement in the truth value judgments was found across ages, it is unclear whether this followed from gains in the ability to process accented speech or simply the ability to perform the task well.

### Effects of exposure

Although no work has experimentally isolated the effects of exposure to natural accents, some research using laboratory learning of artificial accents suggests that children can learn to map ambiguous sounds to specific categories when provided with sufficient information. In McQueen et al. (2012), 6- and 12-year-olds learned to map an ambiguous sound between /f/ and /s/ onto one of these endpoints after hearing them in the context of unambiguous lexical items (such as *platypus* and *giraffe*). Other work suggests that there may be some developmental differences in the ability to integrate multiple cues in order to perform such remapping. van Linden and Vroomen (2008) presented 5- and 8-year-olds with videos where talkers said /aba/ (or /ada/) when the paired audio was an ambiguous sound between /b/ and /d/, and videos of /ada/ (or /aba/) with an unambiguous audio. As adults had in a previous study (Bertelson et al., 2003), 8-year-olds clearly learned to interpret the ambiguous sound in terms of the visually presented category. In stark contrast, 5-year-olds showed no such recalibration.

### Summary

Children are remarkably good at spotting accents, although they may be better with some accents than others (e.g., Floccia et al., 2009). During childhood, the ability to retrieve meaning from accented speech improves with age (Nathan et al., 1998). Short-term exposure to an accent with a single sound change guided by visual or lexical information clearly shapes children’s perception, although there may be developmental changes in the ability to profit from bootstrapping information (van Linden and Vroomen, 2008). Surprisingly, no work has assessed the effects of long-term exposure to an accent in childhood, although clearly this must matter in view of effects on production (Tagliamonte and Molfenter, 2007). It is unclear whether similar mechanisms are used by children and young adults, or whether adaptation strategies vary with cognitive and linguistic development. This seems an unfortunate state of affairs. The empirical and theoretical contribution that research with children has made to our understanding of the development of language *production* is simply extraordinary. There is every reason to believe that it will be just as insightful to evaluate the development of *perception*, as in childhood there are dramatic changes in lexical and cognitive development, which could play a role in accented speech perception.

## Accent Perception in Late Adulthood

Research is increasingly turning to how older adults cope with dialectal, foreign, or simply novel accents, a question that is both theoretically and empirically important. There are several factors which change with aging that could impact accented speech perception. To begin with, older adults often suffer from age-related hearing loss (presbycusis), which impairs sensitivity (i.e., loudness), and fine tuning (i.e., spectral resolution). This hearing loss may render speech perception in general more difficult. It could potentially decrease the difficulty gap between accented and unaccented speech, as it leads listeners to rely on context more. Alternatively, it could also increase the challenge of processing unusual speech patterns, because less information is available. Aging also impacts cognitive function, including speed of processing, working memory, long-term memory, and inhibition or cognitive control (a recent review in Park and Reuter-Lorenz, 2009). Accented speech imposes both linguistic and cognitive load challenges for the listener (see Concluding Remarks for further discussion). The confluence of a reduction in signal strength (due to the listener’s presbycusis), and a reduction in cognitive function (which may be more evident in harder tasks; i.e., for accented speech more than unaccented speech) could lead to significant impairments when aging adults are faced with unusual accents. Understanding how older adults process accented speech thus sheds light on the factors involved in dealing with such variation in the spoken signal, and could provide us with cues to facilitate communication for older adults.

### Initial processing costs

Some previous work has reported that older adults experience greater difficulty when faced with multiple talkers (Sommers, 1996), fast speech (Janse, 2009), and speech in noise (e.g., Kalikow et al., 1977) than younger adults. Thus, one could expect older adults to experience even greater difficulties than younger adults when presented with accented speech. However, most work focusing on this question confirms that while adults do have greater difficulty with accented than unaccented speech, the size of this effect is not significantly greater for older than younger listeners (Burda et al., 2003; Shah et al., 2005; Ferguson et al., 2010; Gordon-Salant et al., 2010a). It does not appear that the absence of a difference across age groups could be due to a ceiling effect (Adank et al., 2009; put forward a similar argument to explain data collected from younger listeners). Indeed, while the addition of multi-talker babble yielded age differences in one study (Gordon-Salant et al., 2010b), it did not in another (Ferguson et al., 2010).

Several factors could explain differences in results like this one. First, perhaps different studies sample from a diverse population without considering variables that would structure this variability. Subsequent work has more explicitly attempted to control for differences in hearing status (e.g., Burda et al., 2003; Ferguson et al., 2010), which is usually found *not* to interact with accented speech perception (but see Gordon-Salant et al., 2010b for a more complex pattern of results; and Janse and Adank, 2012, for contrary results). However, there is still some progress to be made in understanding the effects of cognitive decline, and its contribution to the diversity of results reported. Based on individual variation data, Janse and Adank (2012) report that memory subsystems play a role in accented speech processing. Finally, it may be the case that different results are partially due to differences in the stimuli, particularly the quality of the accent under study or the amount of familiarity with it.

### Effects of exposure

Work on adaptation to altered speech, such as time-compressed or noise-vocoded signals, suggests that older adults definitely profit from short-term exposure to such degraded signals, although the extent to which they resemble younger adults is still a matter of debate (there was no difference across groups in Golomb et al., 2007; in Peelle and Wingfield, 2005, older adults adapted to time-compressed and noise-vocoded speech similarly to younger adults, although the former showed smaller maintenance and transfer effects). More general research on implicit learning and aging suggests that older adults may adopt compensatory strategies, which are often sufficient, except for particularly challenging tasks (for a recent review, see Rieckmann and Bäckman, 2009).

Overall, studies on short-term exposure to accented speech support the view that older adults can adapt to novel speech patterns after brief exposure, but, as with perceptual learning in general, some report differences between older and younger listeners (Adank and Janse, 2010 reported that older listeners stopped adapting more quickly than younger ones); and others find no significant differences across age groups (Gordon-Salant et al., 2010). As argued above, diverse results could be explained by sampling from a variable population. Janse and colleagues have begun investigating whether individual variation in accented speech comprehension and adaptation correlates with individual variation along cognitive and linguistic dimensions. For example, Janse and Adank (2012) report that both measures of selective attention and vocabulary predicted adaptation in a group of older adults. Moreover, while some indices of executive function can predict adaptation, they might not be the same in younger and older adults (Jesse and Janse, 2012). This is clearly a promising avenue of research, which could inform our understanding of the variety of mechanisms that are involved in both accent perception and adaptation.

### Summary

Although only a handful of studies have been carried out with older adults, it is clear that this population experiences an initial cost when processing accented speech, which may be rendered smaller through exposure. But it is still unclear whether this cost is greater or smaller than that found in younger adults, and whether adaptation occurs at the same or a different pace, with the same or different mechanisms. The greatest contribution of the research reviewed in this section centers on the study of factors that structure individual variation in performance. Recent results suggest that accent perception, at least in this population, is greatly affected by linguistic (e.g., vocabulary size) and cognitive (e.g., executive control) dimensions. At this point, however, it remains difficult to determine to what extent initial processing costs and effects of short-term exposure are qualitatively different (i.e., recruit a different profile of speech perception mechanisms) in younger and older adults. As with research in childhood, we feel that this is an understudied population, and hope future work will shed further light on these already interesting results.

## General Discussion

### General points of convergence and divergence across multiple age groups

Specialists in infancy, childhood, adulthood, and aging have all investigated initial processing costs, although the level of precision with which these questions have been investigated varies. The exact ways in which an accent trumps speech processing have been best described for younger adults. Accent affects both speed and accuracy, and may lead to different processing styles in young adults. As for adaptation, it is clear that individuals of all ages can learn to adapt to new accents.

In terms of the mechanisms recruited, lexical feedback is clearly the main source of information that learners have been assumed to use, and the focus of attention has been on single segmental changes. However, infants can adapt to new accents when they are too young to have a large lexicon; and they can do so without a disambiguating lexical context. These facts should inspire adult researchers to consider other aspects of accent processing. We predict that accent adaptation, particularly in infancy, can be triggered by suprasegmental deviations. The presence of such deviations would invite listeners to employ processing schemes that are robust in the face of uncertainty; for example, they should allow less strict acoustic matching and combine more cues for segmentation. We state that such suprasegmental deviations must be crucial triggers of adaptation because young infants have not yet established their native phonological inventory, but we expect that the importance of such “suprasegmental cues to accentedness” may decline with age and experience. In contrast, it is to be expected that lexical factors play an increasingly large role throughout toddlerhood and later childhood, as lexical growth allows listeners to detect accents through mismatches between the original and expected lexical forms. An interesting question, to which the answer is far from obvious, is whether the influence of suprasegmental and lexical cues to accentedness could be a stable predictor of individual differences in accented speech perception in older adults.

One recurrent question was the format of adaptation. While some suggest that learners extract prelexical patterns, others favor lexical storage as the way in which learners capture their newly gained accent knowledge. We have reviewed evidence that 19-month-olds exposed to an artificial accent did not accept *any* sound change in untrained items, but only mispronunciations along the lines of the experienced sound change (White and Aslin, 2011). However, this may not indicate that phonemic remapping is already perfect at this young age. For example, van Linden and Vroomen (2008) suggest that additional experience helps learners become more informed listeners, allowing them to integrate multimodal information.

Furthermore, the work carried out with older adults suggests that certain cognitive skills (such as selective attention) could play a major role in adaptation. It is crucial to extend this insight to other populations, and particularly to infancy, toddlerhood, and childhood, where cognitive skills are in constant development. A good approach would consider the linguistic factors discussed in the previous paragraphs, as well as precise cognitive constructs, in the description of the system underlying perception of deviant speech patterns, rapid short-term adaptation, or gain of novel processing strategies, and long-term changes in a multi-varietal context.

Finally, while hearing status has not been found to impact processing in older adults, it has not been explored in any other age group. Necessarily, having a distorted or smaller signal could have a much greater impact in infancy and childhood, and interact in more complex ways with cognitive skills than it does in older adults. Additionally, future work should examine special populations, such as autistic spectrum disorders (ASDs), Williams Syndrome, and Specific Language Impairment (SLI). Individuals with ASDs are characterized by deficits in social interaction and suprasegmental processing, SLI is characterized by deficits primarily in language processing, and children with Williams Syndrome show a good ability to navigate social territory, yet low IQ scores. This work could shed unique light on the influence of certain social, cognitive, and linguistic factors on accented speech perception, in addition to making steps toward the study of speech perception by *all* language users, and not only normative ones.

One aspect of accented speech perception that we have not covered pertains to links between speech and social evaluation. Listeners engage in social processing during the course of speech recognition, since processing indexical information is not independent from processing of linguistic information (e.g., Mullenix and Pisoni, 1990). An unfamiliar accent has been found to trigger negative social evaluations in infants (Kinzler et al., 2007), children (Kinzler et al., 2009, 2011), and young adults (Preston, 2003; Bresnahan et al., 2002; Lev-Ari and Keysar, 2010; of course, accents that differ from one’s native accent can also have positive or neutral connotations, e.g., van Bezooijen, 2005). Even if the listener has no previous experience with an accent, and therefore cannot evaluate it based on its social valence, speech streams that are harder to process are not as well-liked (Alter and Oppenheimer, 2009). Since accented speech is more difficult to process, it could be routinely disliked at an implicit level. In the confluence of this added linguistic and social processing, accented speech must also differ from non-accented speech in terms of the cognitive load imposed on the listener, a factor that, at least theoretically, may be separable from the complexity of accent marking on the signal (Mattys et al., 2009). Furthermore, this cognitive load may have the greatest effect on populations who have difficulty following explicit instructions (e.g., infants and toddlers) and those with executive control issues (e.g., a subset of the aging population), but it may affect all listeners to a smaller or greater extent. A fuller discussion of the social perception of accent across development awaits a separate review.

### Open questions

While the body of work on accent perception carried out in the past 40 years is noteworthy and solid, there are many areas that necessitate further exploration. Our review of the literature also revealed two recurring “beliefs” not mentioned above, but which are worthy of exploration because they permeate the literature. Ultimately, the most relevant evidence to support these “beliefs” is currently lacking, so we address them here in the hope of inspiring the community to revisit them.

### Processing foreign and within-language accents is fundamentally different

In the Introduction, we merely stated that we would use “linguistic variety” as an umbrella term. We viewed this umbrella as necessary for both conceptual and empirical reasons. At a conceptual level, it is impossible to draw stable, non-arbitrary boundaries between (1) different languages; (2) different dialects of the same language; and (3) non-native, dialectal, sociolectal accents. For example, among linguists, it is often said that “a language is a dialect with an army and a navy” (Magner, 1974). This phrase captures the fact that variation occurs along a continuum, whereas hard boundaries are derived from political, social, and historical reasons, rather than true linguistic distance between two linguistic varieties. At an empirical level, it is not rare to find two “languages” that are closer to each other (in terms of mutual intelligibility and ease of processing) than two “dialects” of the same language. One often cited example is Dutch and German, intuitively conceived as two languages in spite of the fact that they are fairly mutually intelligible; in contrast, Taishanese and Pinghua (“dialects” of Cantonese, itself a “dialect” of Chinese) are not mutually intelligible.

Similarly, it is impossible to describe foreign accent as *always* being more distinct from the listener’s native language than an unfamiliar within-language accent. A common misconception is that dialects differ only in sound instantiation, but not suprasegmentally, whereas two languages will be different both at the segmental and the suprasegmental level. This is simply not the case. For instance, Dutch is rhythmically more similar to Standard Southern British (SSB) than Glaswegian English (White et al., 2012); therefore, it could be easier for SSB-learning infants to segment words in Dutch or Dutch-accented English than in Glaswegian English. Naturally, SSB adults will have a harder time understanding a Dutch speaker than a fellow Glaswegian, given the smaller lexical overlap with the former variety. In other words, it is not always the case that dialects are closer to each other than languages. Moreover, the degree to which *processing* an unfamiliar within-language accent resembles processing an unfamiliar foreign accent at any given age is an empirical matter and probably depends on the dimension of focus.

And yet, it is extremely common to find statements concluding that non-native accents are fundamentally different (e.g., Major, 2007; Adank et al., 2009; Munro et al., 2010; Weber and Poellmann, 2010; Goslin et al., 2012). In one sense, the statement is trivially true: people who are speaking a language that is not their native one will almost certainly be under greater cognitive and motor strain than those speaking their native language, which will cause a host of paralinguistic acoustic changes (such as disfluencies and increased variability in target production) above and beyond the “interesting” linguistic deviations. But in another sense, the statement is simply misleading. We would like to invite the research community to remove from the table the arbitrary distinctions language/dialect/foreign accent, and replace them by (psycho) linguistic distance metrics. These metrics should consider not only rhythmic, intonational, phonetic, phonological, and lexical similarity between the specific speech samples being used; but also the additional cognitive and/or social costs brought about by those changes, and which are dependent on certain characteristics of the listener group under study, ranging from degree of experience with the variety to social values associated with it. Naturally, this renders our descriptive task considerably more difficult, since these distance metrics will necessarily vary across ages, linguistic backgrounds, and possibly even tasks. Nonetheless, we will hopefully gain in explanatory power, as those distance metrics will be available to listeners either as cognitive constructs, or as ready-made distinctions on the signal, whereas the arbitrary “accent” bins are likely not. A first step along this direction could make use of the masses of knowledge already accumulated, regressing infant, child, and adult perceptual measures on a host of possible measures calculated from the actual stimuli used. Once candidate measures are thus identified, they can be more neatly investigated in *ad hoc* experimental studies.

The question of how to draw lines between linguistic varieties is relevant for another line of research. It has been repeatedly reported that bilingual speakers develop more flexible cognitive and linguistic systems (Kovacs and Mehler, 2009; Bialystok, 2010; Sebastián-Gallés, 2010). If the line between accents, dialects, and languages is difficult to draw, does this mean that bi-accentual/bi-dialectal children will also experience similar cognitive gains? The degree of similarity in cognitive gains exhibited by bilingual listeners and those with exposure to two within-language accents is likely dependent on the similarity in their life experiences. That is, children who are routinely exposed to two variants, but grow up to speak only one variant may experience lower internal conflict and require lower strength of inhibition than those who come to speak two variants. This becomes particularly relevant when considering an infant who is trying to learn the sound system of the language(s) she hears, and may not know whether she is hearing one or multiple varieties of the same or different languages (for recent summaries on infant bilingual acquisition, see Sebastián-Gallés, 2010; Werker, 2012). For example, one may argue that it should be more difficult to tease apart Spanish from Catalan, which are very similar at the phonological level, than native English from a heavily French-accented English, since French differs from English even at the rhythmic level. Thus, perhaps the degree of *perceptual* difference between the varieties might also be a factor, even when the child grows up to speak only one variety. Although we cannot attempt it here, we hope a future review will explore similarities and differences between bi-accentual/bi-dialectal and bilingual acquisition.

### Processing different accents and different voices is fundamentally the same

Even encountering novel talkers within one’s own accent group presents the perception system with massive inter-speaker variation, which has a processing cost. Listening to speech by multiple talkers as compared to one talker results in slower reaction times and disrupted accuracy on many tasks, a phenomenon that has been called the *talker interference* effect (Creel and Bregman, 2011). For example, listeners are slower to respond in a word monitoring task when there are multiple talkers than when there is only one talker (e.g., Nusbaum and Morin, 1992 for words; Assmann et al., 1981 for vowels), and this slowing is affected by working memory load (Nusbaum and Morin, 1992). Likewise, when given a set of utterances, listeners are slower and less accurate at naming a word spoken in noise if the utterances are spoken by a mix of talkers instead of one talker (e.g., Creelman, 1957; Mullennix et al., 1989; Sommers et al., 1994). Finally, listeners recall fewer words from a list spoken by multiple talkers as compared to a list spoken by one talker (Martin et al., 1989, but see Goldinger et al., 1991 and Nygaard et al., 1994 for evidence that inter-stimulus-interval modulates this effect). To a certain extent, the talker interference effect is due to top-down biases, since it emerges when the listeners *expect* to hear two voices, even if the signal from “both voices” is acoustically identical (Magnuson and Nusbaum, 2007, using synthetic speech).

Behavioral measures of multi-talker speech perception reveal not only a processing cost, but also phenomena that can be compared to the short-term perceptual adaptation effects and long-term effects of exposure noted above. Akin to accent adaptation, repeated exposure to a given talker aids speech processing in a variety of tasks. First, word recognition under difficult processing conditions or in the presence of noise is enhanced when the listener has some experience with the talker (Nygaard et al., 1994; Nygaard and Pisoni, 1998). Recognizing spoken-words in noise is facilitated even when the familiarization phase only involves lip-reading (Rosenblum et al., 2007). Second, voice familiarity appears to increase memory for words or sentences, though results vary for different task types (see Luce and Lyons, 1998; Goh, 2005, for discussions). Recognition memory for words is more accurate when the voice is the same at exposure and at test, and this same voice priming can last for up to a week (Goldinger, 1996). Similarly, with a continuous recognition memory paradigm, listeners are better at recognizing whether a given word was previously heard if the second presentation of the word is in the same voice as the first presentation rather than in a different voice (Palmeri et al., 1993; Bradlow et al., 1995; see also Martin et al., 1989; Goldinger et al., 1991 for similar effects of talker variability/consistency on serial recall). The effects of voice consistency appear to be more robust in explicit memory tasks than in implicit memory tasks (Goldinger, 1996; Luce and Lyons, 1998; but see Schacter and Church, 1992 and Church and Schacter, 1994 for evidence of voice effects in implicit tasks).

Additionally, in many perceptual adaptation paradigms, listeners are exposed to a single talker with a quirky pronunciation, and tested on the same voice used in the exposure phase. Therefore, unless follow-up experiments are carried out, it usually is impossible to be sure that results reflect adaptation to very specific features, or rather more general adaptation processes. In fact, when the question has been addressed, it has been found that the level of specificity varies across studies. Indeed, in some studies, listeners interpret the remapping as being *situation-specific* (e.g., the talker had a pen in her mouth, hence she does not normally talk funny; Kraljic et al., 2008b); in others, listeners interpret it as being *talker-specific* (a quirky /s/ in the speaker’s non-native language is expected to remain quirky in the speaker’s native language; Reinisch et al., 2012); and yet other studies document *talker-general* patterns of adaptation (retuning to stop voicing, but not fricative place, is generalized across talkers; Kraljic and Samuel, 2007).

Thus, based on the similarity of behavioral evidence of initial processing costs and perceptual adaptation effects, one could posit the hypothesis that the same *underlying mechanisms* govern processing of talker and accent variation (e.g., Nygaard and Pisoni, 1998). In some cases, the parallel between voice and accent normalization is transformed into an explicit assumption of how speech processing should proceed. In most infant/toddler accent perception studies, it is assumed that children should come to ignore talker accent just as they ignore voice (see e.g., Best et al., 2009). For example, a similar developmental timeline has been posited for the two in word segmentation tasks (see Schmale and Seidl, 2009; Schmale et al., 2011, for further discussion).

Although the parallels between processing talker and accent variation are remarkable, further work is needed before concluding that this stems from their involving the same mechanisms. To begin with, some of the changes imposed on the signal by a talker’s voice are due entirely to their physical properties, which are automatically undone by our auditory system with little need for linguistic representations (see e.g., Lotto et al., 1997, for a summary of animal research on talker normalization). It is unlikely that differences between linguistic varieties can be undone through such universal and innate mechanisms. Additionally, while all children have some exposure to talkers with different voices, not all children have exposure to multiple accents. Thus, infants have positive evidence of the kinds of additional transformations that are required to deal with multiple talkers, but may not have developed robust remapping mechanisms for different accents. Thus, it is an empirical matter as to what extent the mechanisms recruited, at any given age and for a given task, are overlapping for talker and accent variation.

### Concluding remarks

This review reveals some points of convergence of research on accent perception across the lifespan. Throughout the lifespan, online measures have provided evidence that an accent can initially impair linguistic processing, but further experience allows for rapid adaptation. Admittedly, obtaining a full picture of the development of accented speech perception from infancy to adulthood is impossible at present, especially given the major methodological and theoretical differences that exist across research with infants, children, and adults. In this quest, it will be necessary to develop appropriately controlled stimuli, and to establish which behavioral and brain measures are comparable across populations. Ultimately, it would benefit researchers to employ comparable tasks that can be implemented across the lifespan. This type of methodological innovation would allow researchers to more reliably identify specific developmental changes in accent perception.

The second set of roadblocks can be argued to relate to theoretical factors. First, it is likely impossible, and arguably unnatural, to design tasks which isolate a single dimension of interest, such as the effect of linguistic deviations while controlling for social and cognitive effects. Additionally, when assessing populations as different as infants and adults, we simply cannot assume that the linguistic system is organized in the same way. For example, infants may not parse speech into phone-sized categories that are limited to the native inventory, assigning them symbolic labels. Certainly, these theoretical challenges complicate the interpretation of findings within the realm of infant and child research, and render it more difficult to draw inferences from research carried out at many ages across the lifespan.

Nonetheless, individuals in all age groups grapple with accented speech. Therefore, research on accented speech perception makes a unique contribution to our understanding of ecologically valid language processing. Over this backdrop, the contribution of the present article relied on the comparison of research carried out at different points of the lifespan. This comparison both uncovered the aspects of linguistic processing that are common to all human perceivers and underlined which aspects can vary across individuals and populations.

## Conflict of Interest Statement

The authors declare that the research was conducted in the absence of any commercial or financial relationships that could be construed as a potential conflict of interest.
